# Critical timing of ACEi initiation prevents compensatory glomerular hypertrophy in the remaining single kidney

**DOI:** 10.1038/s41598-021-99124-z

**Published:** 2021-10-01

**Authors:** Abhijit S. Naik, Su Q. Wang, Mahboob Chowdhury, Jawad Aqeel, Christopher L. O’Connor, Jocelyn E. Wiggins, Markus Bitzer, Roger C. Wiggins

**Affiliations:** 1 Department of Internal Medicine, University of Michigan, Ann Arbor, MI USA; 2 F6676 UHS, 1500 E Medical Center Dr, Ann Arbor, MI 48109-5676 USA; 3 1570B MSRB2, 1150 W Medical Center Dr, Ann Arbor, MI 48109-5676 USA

**Keywords:** Nephrology, Glomerular diseases

## Abstract

Increasing evidence suggests that single in kidney states (e.g., kidney transplantation and living donation) progressive glomerulosclerosis limits kidney lifespan. Modeling shows that post-nephrectomy compensatory glomerular volume (GV) increase drives podocyte depletion and hypertrophic stress resulting in proteinuria and glomerulosclerosis, implying that GV increase could serve as a therapeutic target to prevent progression. In this report we examine how Angiotensin Converting Enzyme inhibition (ACEi), started before uninephrectomy can reduce compensatory GV increase in wild-type Fischer344 rats. An unbiased computer-assisted method was used for morphometric analysis. Urine Insulin-like growth factor-1 (IGF-1), the major diver of body and kidney growth, was used as a readout. In long-term (40-week) studies of uni-nephrectomized versus sham-nephrectomized rats a 2.2-fold increase in GV was associated with reduced podocyte density, increased proteinuria and glomerulosclerosis. Compensatory GV increase was largely prevented by ACEi started a week before but not after uni-nephrectomy with no measurable impact on long-term eGFR. Similarly, in short-term (14-day) studies, ACEi started a week before uni-nephrectomy reduced both GV increase and urine IGF-1 excretion. Thus, timing of ACEi in relation to uni-nephrectomy had significant impact on post-nephrectomy “compensatory” glomerular growth and outcomes that could potentially be used to improve kidney transplantation and live kidney donation outcomes.

## Introduction

The 5/6 nephrectomized (Nx) rat has long served as a convenient model of autonomously progressive glomerular disease that reaches ESKD over weeks. Brenner, Hostetter and colleagues used this model to define the hyperfiltration hypothesis and identify ACEi as protective against glomerular disease progression that now serves as the mainstay for preventing glomerular disease progression in the clinic^1–3^. Uni-Nx (3/6 nephrectomy) in rats also induces autonomous development of proteinuria, glomerulosclerosis, and ESKD, but only after a period of apparent stability manifest by increased protein excretion reflecting ongoing hyperfiltration and compensatory glomerular and kidney enlargement. In uni-nephrectomized humans larger nephron size is associated with worse long-term outcomes^4^. This raises the question of whether single kidney states should be viewed as metastable and at risk of reduced kidney lifespan absent hypertrophic stress prevention.

Although living human kidney donors did not manifest significant renal function loss after ten years, follow up over 15–20 years is associated with increased CKD risk, particularly in a setting of superimposed hypertension or diabetes^5–9^. Furthermore, a large meta-analysis of living donors shows higher urine protein excretion rates compared to controls that increases with time after donation suggesting ongoing progressive glomerular stresses^10^. Kidney donors are often healthy young individuals with expectation of a long post-donation lifespan and CKD risk increases exponentially with time after nephrectomy^11^. Therefore strategies that could predict and mitigate this risk are needed.

In parallel to increased kidney donor risk, several lines of evidence suggest that long-term (as opposed to short-term) allograft attrition is also caused by progressive glomerulosclerosis. Two long-term protocol biopsy studies show that the predominant pathologic process associated with the worse long-term outcome is global glomerulosclerosis^12,13^. All allografts hyperfilter to a greater or lesser extent^14^. Therefore, as suggested by Brenner and colleagues, allografts are susceptible to developing hyperfiltration-associated glomerulosclerosis^2^. Compatible with this concept, smaller donor body/kidney size in relation to larger recipient body/kidney size is associated with worse allograft outcome and vice versa*,* implying that hypertrophic stress plays a significant role in graft outcome^15,16^. All allografts have an increased rate of podocyte detachment detectable in the urine that will predispose to progressive glomerulosclerosis^17–19^. The amount of podocyte loss in the first year post-transplantation is related to the kidney donor to recipient body size ratio with smaller donor kidneys transplanted into larger recipients having more podocyte detachment and vice versa^18^. Furthermore, the degree of podocyte detachment is predictive of the rate of loss of long-term allograft function^18^. A recent report suggests a significant benefit of angiotensin II blockade in allografts if initiated early after transplantation, a finding also supported by a large systematic review^20,21^. These data indicate that hypertrophic stresses incurred at transplantation could result in increased podocyte stress and detachment leading to progressive glomerulosclerosis and contributing to shortened allograft lifespan.

Ichikawa and colleagues first described the link between glomerular hypertrophy and sclerosis^22–24^. Consistent with this concept, we demonstrated that glomerular growth itself could trigger progressive glomerulosclerosis by employing a transgenic uni-nephrectomised rat model whose podocytes were engineered to have impaired mTORC1-driven protein synthesis in response to growth factor and nutrient signaling^25–27^. In this model angiotensin-converting enzyme inhibition (ACEi), that reduces hyperfiltration, also reduced glomerular and kidney growth in the remaining kidney. It implied that the prevention of peri-nephrectomy hemodynamic changes in the remnant kidney were causally related to reduced compensatory kidney hypertrophy and glomerular enlargement. Further support for this concept comes from Sigmon and colleagues who showed that preventing post-Nx hyperfiltration in the remnant kidney by inhibiting nitric oxide (NO)-induced vasodilation using the NO synthase inhibitor L-NAME (N(ω)-nitro-L-arginine methyl ester) prevented kidney and glomerular compensatory hypertrophy in the remaining kidney^28^. Thus, two independent approaches support the hypothesis that, following uni-Nx, changes in remaining kidney hemodynamics leads to hyperfiltration of factor(s) from the blood that drives kidney and glomerular hypertrophy. This provides a hypothetical construct by which peri-nephrectomy hemodynamic changes caused by transitioning from the two kidneys to the one kidney state could initiate and drive progressive glomerular disease. Furthermore, it raises the question of whether starting ACEi prior to uni-nephrectomy might provide additional benefit compared to starting ACEi after nephrectomy.

Post-natal coordinated body and organ growth is regulated through insulin-like growth factor-1 (IGF-1) under the control of pituitary-derived growth hormone (GH)^29,30^. Substantial evidence from prior literature suggests that IGF-1 also plays a vital role in driving both compensatory kidney hypertrophy and glomerular enlargement^31^, including IGF-1 receptor blockade preventing compensatory kidney hypertrophy^32^, These data suggest that hyperfiltered IGF-1 may drive compensatory kidney hypertrophy and, therefore, that reduction of IGF-1 hyperfiltration by ACEi would also reduce compensatory kidney and glomerular growth. We tested this hypothesis using a rat uni-Nx model and found that starting ACEi > 7 days before uni-Nx (but not after uni-nephrectomy) markedly reduced both IGF-1 hyperfiltration and compensatory glomerular hypertrophy.

## Materials and methods

Wild-type Fischer344 rats (purchased from Charles River Inc., Wilmington, MA) were used for the study since they are relatively resistant to developing diabetes or hypertension. Animal health monitoring was provided daily by the University of Michigan as approved by the Institutional Animal Care & Use Committee (IACUC) of the University of Michigan under #PRO00006209. All experiments were performed in accordance with relevant guidelines and regulations. The study was carried out in compliance with the ARRIVE guidelines (https://arriveguidelines.org).

Rats that were losing weight and/or had decreased appetite or high-volume urine output were closely monitored and euthanized if they had progressive signs and symptoms of kidney failure.

### Rat uninephrectomy model

Male rats underwent uninephrectomy or sham uninephrectomy at 300 g or 100 g body weight interventions as described that lasted for 2 days to 10 months. Rats were weighed daily or once per week for shorter-term studies and once per month for long-term studies. For timed overnight urine collections, rats were placed into a metabolic cage. Urine and plasma creatinine were measured by the alkaline picrate method using a plate assay. Urine and plasma protein concentrations were measured by TCA precipitation followed by solubilization of the precipitate in NaOH and Bradford plate assay. In the nephrectomy/sham-nephrectomy study systolic blood pressure was measured by tail cuff at the end of the study. For the ACEi study systolic blood pressure was measured monthly. Rats were euthanized with ketamine/diazepam, and kidneys were harvested and weighed. Perfusion-fixation of kidneys used PLP buffer (containing paraformaldehyde [2%], lysine [1.37%], and periodate [0.2%] at + 4 °C at 100 mmHg).

### IGF-1, IGF-2, and EGF assays

Rat IGF1 was measured using the R&D Systems ELISA Plate assay (Cat# DY791). IGF-2 was measured using R&D Systems Quantkine ELISA (Cat# MG200). EGF was measured using the R&D Systems Rat ELISA Plate assay (Cat# DY3214). Dilution of urine samples 1:10 and 1:100 in phosphate-buffered saline did not change the measured IGF-1 levels showing that the assay was not affected by other factors present in urine.

### Immunohistochemistry and computer-assisted morphometric analysis

Slides were cut at 3 µm, deparaffinized and rehydrated before incubation in antigen retrieval solution (Retrieve-All Unmasking System 1; Bio Legend 928101, Denham, MA) at 92 °C for 2.5 h. Slides were then blocked with 10% goat serum prior to incubation with either rabbit anti-rat WT1 monoclonal antibody (Abcam A89901) or mouse anti-rat Glepp1 monoclonal antibody 1B4. The detection system used horseradish peroxidase-conjugated rabbit anti-mouse IgG pre-absorbed with goat and rat serum before incubation with the peroxidase substrate (3,3′-diaminobenzidine [DAB]). Slides were then counterstained with periodic acid Schiff and hematoxylin, dehydrated, and mounted. Mounted slides were then digitally scanned at 40× by the Michigan Medicine pathology core facility (Leica Biosystems AT2). True podocyte nuclear density and size were estimated using the quadratic equation to correct for section thickness and nuclear shape using a downloadable spread-sheet^33^. All glomeruli in each rat were qualitatively assessed as being either containing an area of sclerosis however small (designated as “sclerotic”) or not having an area of sclerosis (designated as being non-sclerotic). Fifty of the non-sclerotic glomeruli per case were then randomly selected for morphometric analysis. A computer-assisted image analysis algorithm was used to automatically identify and circumscribe each glomerulus in GLEPP1/PAS-stained sections digitally scanned at 40x. Extracted images were used to estimate glomerular tuft area, %Glepp1-positive area, glomerular volume, podocyte nuclear number, and average podocyte volume^34^.

### Statistical analyses

Rat studies are shown as the Mean ± 1SD. A paired t-test was used for paired studies and unpaired t-test was used for other comparisons. The ANOVA test compared the means of variables in two or more independent groups with Bonferroni correction. Figure 2 contains a nephrectomy group obtained from 2 separate experiments. One where nephrectomy was compared to sham nephrectomy and one where nephrectomy was compared to ACEi taken 8 days before or 4 days after nephrectomy.

## Results

### Long-term uni-nephrectomy studies

#### By 40-weeks after uni-Nx glomerular volume had increased 2.2-fold above that of sham-Nx rats

**Figure 1:**
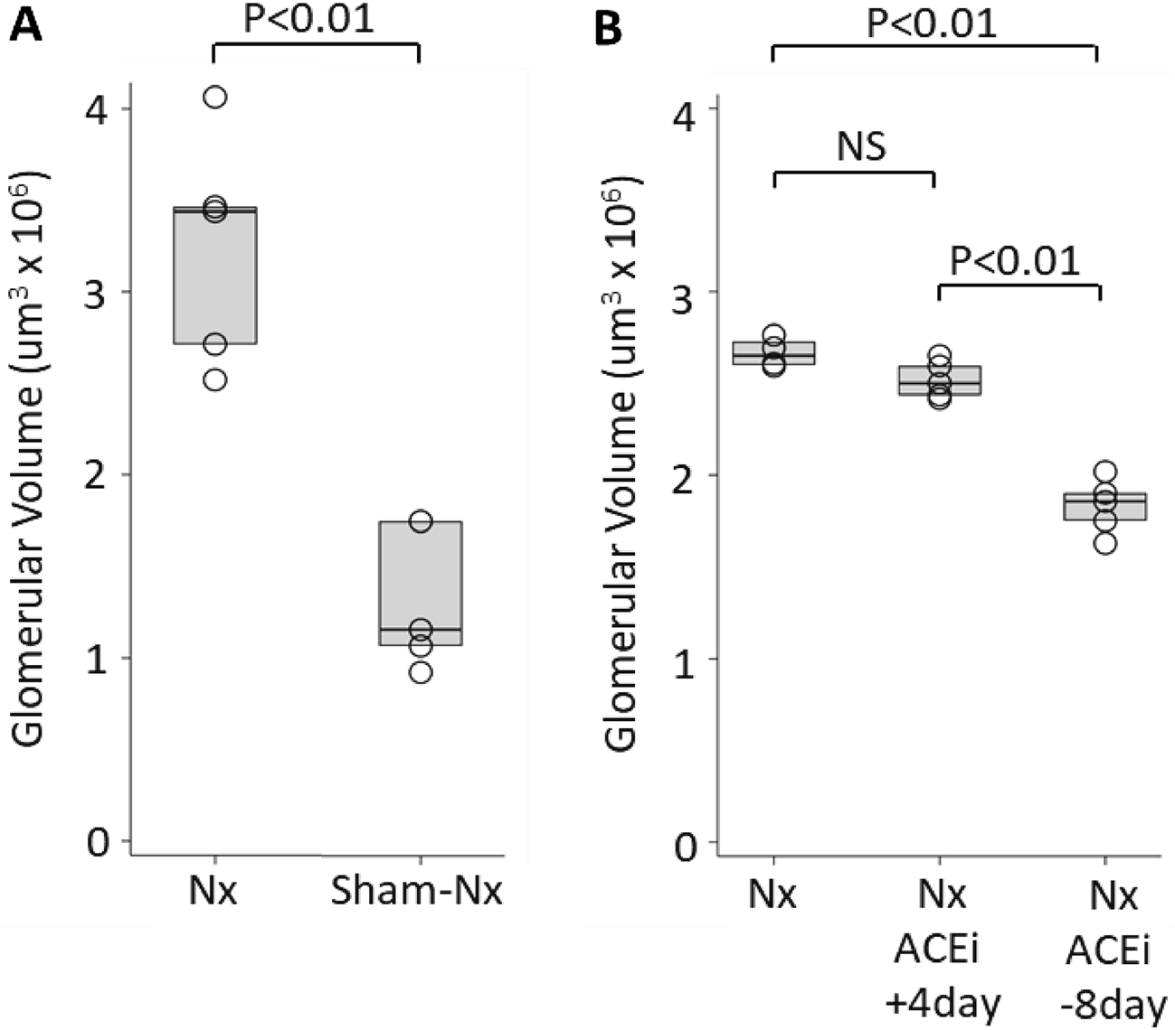
Panel (**A**) Glomerular volume increases by 40 weeks after uni-nephrectomy vs. Sham-nephrectomy. GV had increased 2.2-fold in nephrectomized (one kidney) versus sham-nephrectomized (two kidneys) rats. Nx n = 5, sham-Nx n = 4. Panel (**B**) The effect of ACEi treatment on GV of nephrectomized rats. ACEi treatment (enalapril 10 mg/kg/day delivered in the drinking water) starting 8 days *before* uni-nephrectomy resulted in a significant reduction in glomerular volume, such that GV remained comparable to that of sham-nephrectomized rats shown in Panel (**A**). In contrast, the glomerular volume of rats started on ACEi treatment 4 days *after* uni-nephrectomy increased to a value not significantly different from that of nephrectomized rats that did not receive ACEi. Values for glomerular volume shown are for glomerular profiles in which no glomerulosclerosis was present. Nx n = 4, ACEi + 4 days n = 5, ACEi-8 days n = 5.

**Figure 2:**
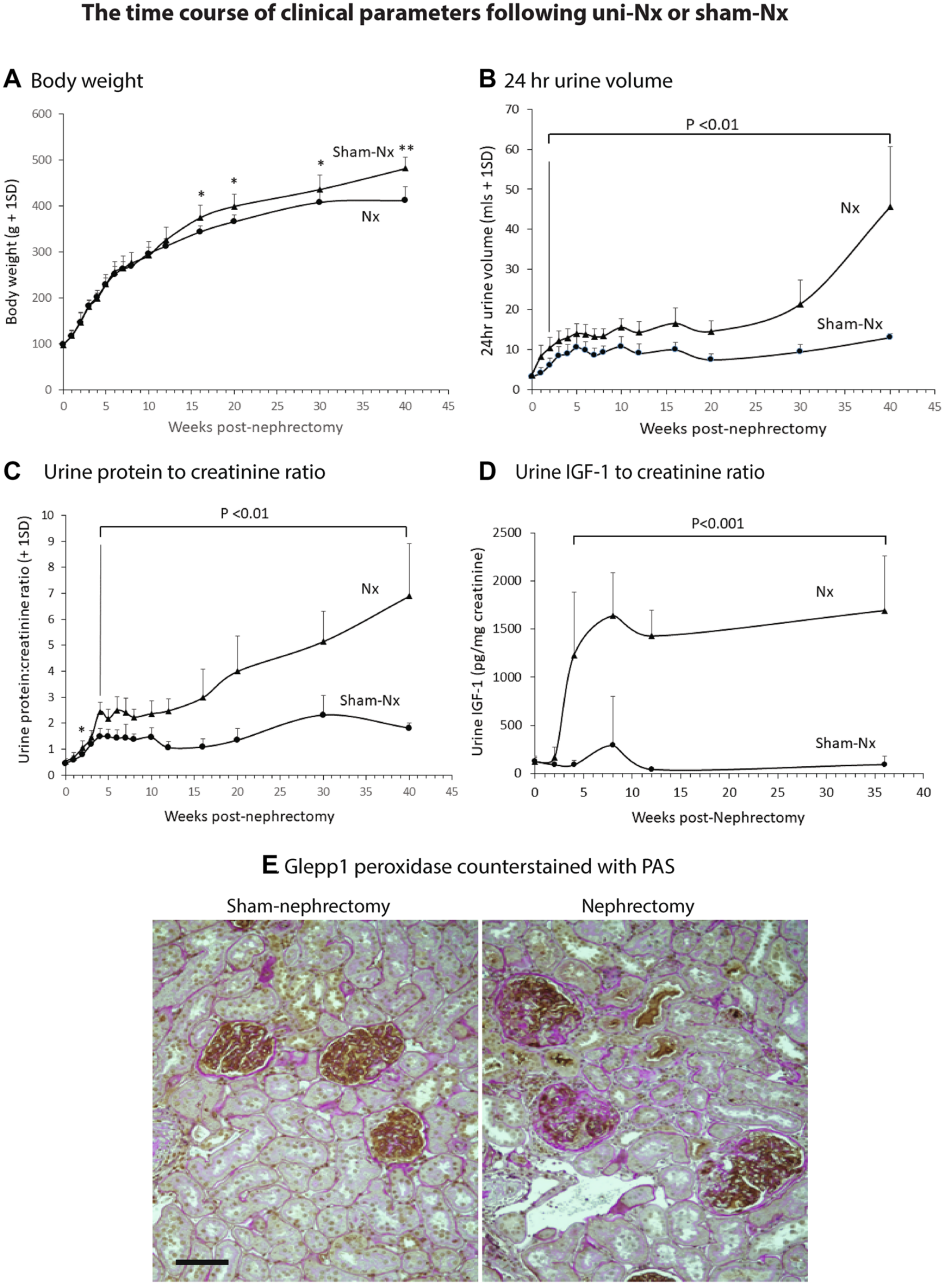
Clinical data for the long-term rat uni-nephrectomy versus sham nephrectomy study. Panel (**A**) Body weight gain. Panel (**B**) 24 h urine volume. Panel (**C**) Urine protein:creatinine ratio. Panel (**D**) Urine IGF-1 to creatinine ratio. Panel (**E**) Histology at 40 weeks post-nephrectomy or sham-nephrectomy. Glepp1 peroxidase (brown) is used to identify podocytes in sections counter-stained with Periodic Acid Schiff (PAS) reagent. Note larger glomeruli in nephrectomized rats together with loss of podocytes (brown) and increased glomerulosclerosis (pink) in glomeruli of uni-nephrectomized rats but not in glomeruli of sham-nephrectomized rats. While 50% of glomeruli had some level of sclerosis only 2.8% of glomeruli were globally sclerotic. Interstitial scarring in the uni-Nx rats is shown by pink staining. The bar represents 50um. *P < 0.05, **P < 0.01.

Figure 1A shows that, by 40-weeks post-surgery, the mean GV of uni-Nx rats (2.98 ± 0.54 um^3^ × 10^6^) was 2.2-fold larger than the GV sham-Nx rats (1.34 ± 0.40 um^3^ × 10^6^), (*P* < 0.01). The time course of clinical parameters following uni-Nx or sham-Nx is shown in Fig. 2. Following uni-Nx rats remained relatively stable for about 15 weeks, albeit with increased urine volume and higher protein excretion compared to sham-Nx rats. However, by 40-weeks post-uni-Nx urine protein excretion had increased to 5-fold, and 50% of glomeruli had developed some degree of glomerulosclerosis and 2.8% of glomeruli were globally sclerotic associated with tubular atrophy and interstitial fibrosis that was not present in the sham-Nx rat kidneys (Fig. 2). Systolic blood pressure measured by tail-cuff at 40-weeks was 136.3 ± 4.4 mmHg for sham-Nx rats and 140.3 ± 2.7 mmHg for Nx rats (NS). Over longer follow-up uni-Nx rats developed increasing glomerulosclerosis and death from uremia by about 15–18 months (data not shown).

#### ACEi reduced uni-Nx-induced glomerular volume increase if initiated prior to uni-Nx

As shown in Fig. 1B, if ACEi (enalapril 10 mg/kg/day delivered in the drinking water) was provided to uni-Nx rats starting eight days *before* uni-nephrectomy and continued long-term, rat GV was significantly prevented from increasing (1.83 ± 0.15 vs 2.67 ± 0.02 um^3^ × 10^6^) (*P* < 0.001), remaining only modestly increased compared to the GV of intact sham-Nx rats shown in Fig. 1A. In contrast, if the ACEi treatment was initiated four days *after* uni-nephrectomy and continued for 40 weeks, then GV increase following uni-Nx was not significantly different from that of non-ACEi-treated uni-Nx rats. Therefore, the timing of ACEi intervention in relation to the uni-Nx event had a highly significant long-term effect on the degree of compensatory GV enlargement by 40 weeks post-uni-Nx.

Table 1 shows that, unlike GV, kidney weight was not significantly different between the two ACEi-treated groups. However, in both ACEi treatment groups, kidney weight, kidney weight:body weight ratio, proteinuria and systolic blood pressure were significantly reduced compared to the non-ACEi-treated group. Notably, the lower GV maintained long-term in rats pre-treated with ACEi did not translate to a significant reduction in measured creatinine clearance (eGFR).

**Table 1:** Comparisons for the effects of ACEi started either 4 days after nephrectomy (+ 4 days) or 8 days before nephrectomy (-8 days) with non-ACEi-treated rats at 44 weeks after uni-nephrectomy.

Treatment	Values at 40 weeks post-nephrectomy		
	a	b	c
	No ACEi	ACEi + 4 days	ACEi − 8 days
n	4	5	5
Body weight (g)	407.8 ± 19.6^ns^	398.2 ± 18.3^ns^	407.8 ± 18.8^ns^
Kidney weight (g)	2.26 ± 0.09**	1.95 ± 0.13^ns^	1.95 ± 0.11**
Kw/Bw (g/Kg)	5.54 ± 0.22**	4.90 ± 0.36^ns^	4.78 ± 0.39**
Glomerular volume (um^3^ × 10^6^)	2.67 ± 0.02^ns^	2.52 ± 0.10**	1.83 ± 0.02**
Podocyte # per glomerulus	166 ± 69**	352 ± 30^ns^	338 ± 17**
Podocyte density (per 10^6^ um^3^)	59 ± 29**	140 ± 14**	185 ± 8**
Sclerotic glomeruli (%)	51.2 ± 9.5**	1.4 ± 2.2^ns^	0.0 ± 0.0**
Systolic blood pressure (mmHg)	133.8 ± 4.5**	100.8 ± 4.7^ns^	108.1 ± 6.3**
Creatinine Clearance (ml/min)	1.07 ± 0.22^ns^	1.30 ± 0.52^ns^	1.29 ± 0.22^ns^
Urine protein (mg/24 h)	120.2 ± 26.5**	18.8 ± 2.0^ns^	17.3 ± 2.0**
Urine IGF-1 (ng/24 h)	76.6 ± 16.4**	3.5 ± 1.6^ns^	2.7 ± 1.8**
Statistical comparisons	a versus b	b versus c	c versus a

The effect of ACEi start time on long-term post-uni-Nx outcome. Starting ACEi 8-days before uni-Nx had no measurable morphometric effect as shown by GV which was 0.47 ± 0.04 um^3^ × 10^6^ in ACEi pre-treated rats compared to 0.46 ± 0.05 um^3^ × 10^6^ or 0.48 ± 0.05 um^3^ × 10^6^ in rats started on ACEi either 4 days after uni-Nx or in non-ACE-treated rats respectively. Similarly, the podocyte number per glomerulus and podocyte density were not different between the group pre-treated with ACEi versus those that were not pre-treated with ACEi.

Thus, ACEi treatment started > 7-days *before* versus 4-days *after* uni-Nx had a significant effect on long-term GV, thereby demonstrating that what happens in the peri-Nx time frame has important long-term consequences. We therefore examined short-term events occurring over the first 14 days post-uni-Nx and the effect of timing of ACEi initiation on these events.

### Short-term uni-nephrectomy studies

#### Post-uni-Nx, remaining kidney hypertrophy is maximal within two days post-uni-Nx and completed by 10 days post-uni-Nx

**Figure 3:**
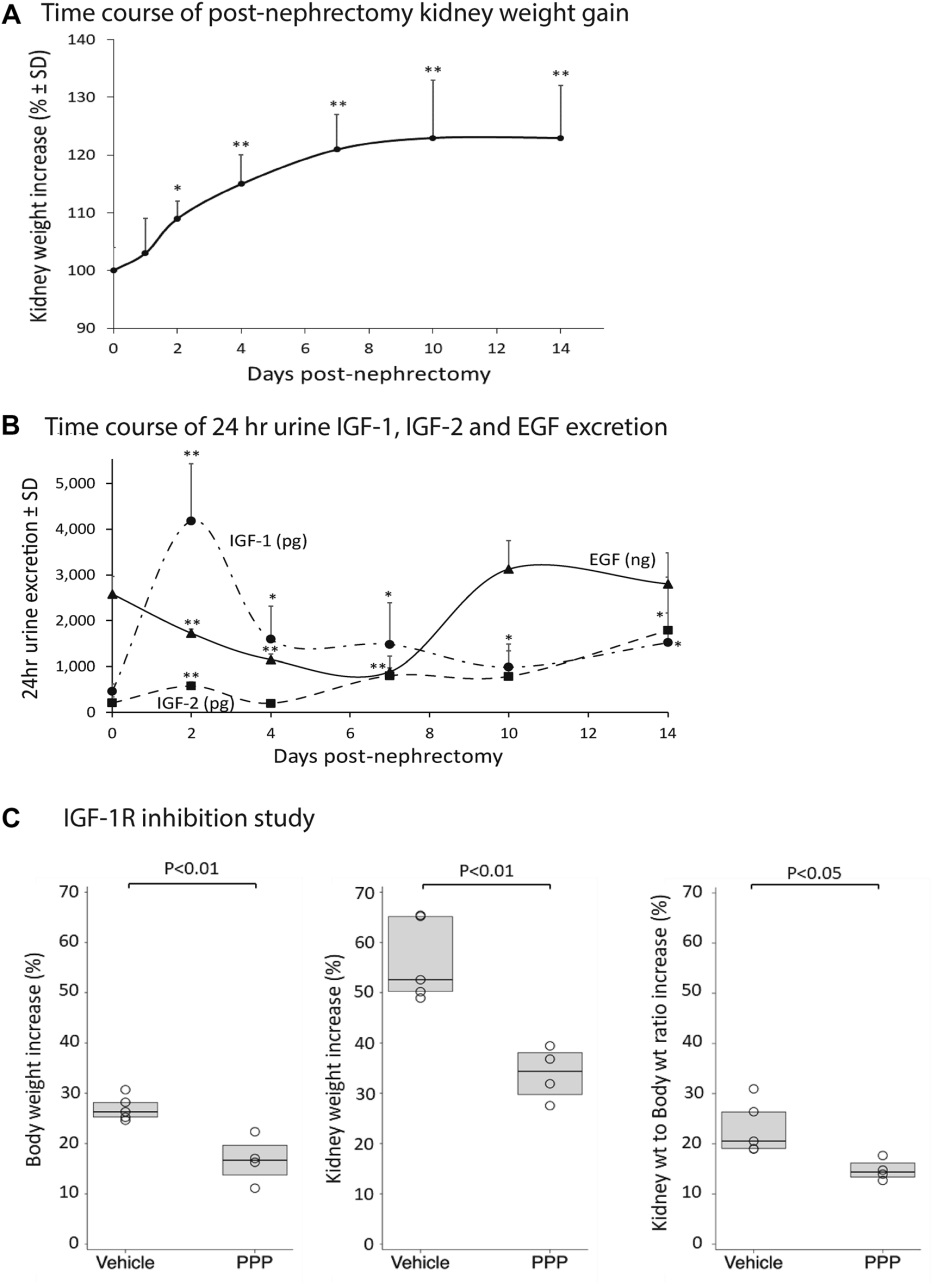
Short-term uni-nephrectomy studies. Panel (**A**) Rapid weight gain of the remaining kidney following uni-nephrectomy in 300 g rats. The kidney growth rate is maximal by 2 days post-uni-nephrectomy and completed by 8-10 days post-uni-nephrectomy (n = 5 per group). *P < 0.05, **P < 0.01 compared to the baseline value. Panel (**B**) Time-course of urine IGF-1, IGF-2, and EGF excretion after uni-Nx. Urine IGF-1 peaks 8-fold above baseline by day-2 post-uni-Nx and remain significantly increased for 14-days. Urine IGF-2 increases 2-fold by day-2 post-uni-Nx but is increased 10-fold by day-14 post-uni-Nx. Urine EGF decreases after uni-Nx but returns to baseline by day-14 post-uni-Nx. Panel (**C**) IGF-1R inhibitor study. The IGF-1R inhibitor picropodophyllin (PPP) was compared to vehicle-injected IP 12 hourly and started 1 day before uni-Nx of 100 g rats and continued for 8-days. PPP reduced body weight gain (left panel), kidney weight gain (middle panel), and kidney weight/body ratio (right panel). The error bars are 1SD. *P < 0.05, **P < 0.01.

Post-uni-Nx, the *rate* of compensatory increase in weight of the remaining kidney was maximal by 2 days (*P* < 0.05) and kidney weight had plateaued 23% above baseline by 8–10 days (*P* < 0.01) (Fig. 3A). To assess whether the increase in kidney weight by 2-days post-uni-Nx was due to kidney water accumulation as a result of hyperfiltration-related factors, we compared the dry weight of kidneys at the time of uni-Nx and 2 days after uni-Nx under the hypothesis that if the kidney weight increase was due to water accumulation then the dry weight as a percent of the total kidney wet weight would be decreased at the 2-day time point. The kidney dry weight as a proportion of wet weight at time of uni-Nx (23.1 ± 0.4%) and 2-days post-uni-Nx (at 23.2 ± 0.2%) (n = 5 per group) were not different. Therefore, the increase in kidney weight observed at 2-days post-uni-Nx was not caused only by additional water accumulation in the remaining kidney, and must therefore reflect the very rapid growth occurring in the remaining kidney by the 2-day time point triggered by uni-Nx.

Since, a primary regulator of compensatory kidney growth is thought to be IGF-1 we evaluated whether increased IGF-1 in urine would be detectable at 2-days post-uni-Nx reflecting increased kidney IGF-1 exposure that could potentially drive the observed increase in kidney and glomerular growth post-uni-Nx.

#### Urine IGF-1 peaks 8-fold above baseline by 2-days post-Nx

The time-courses of IGF-1, IGF-2 and EGF appearing in urine were measured over 14 days following uni-Nx focusing on the day-2 time-point when the kidney growth rate is maximal. Figure 3B shows that by day-2 post-uni-Nx, there was an 8-fold increase in 24 h urine IGF-1, but only a 2-fold-increase in urine IGF-2. By day-14, 24-h urine IGF-1 remained 3-fold significantly above baseline and IGF-2 had increased to 10-fold above baseline. In contrast, 24 h urine EGF decreased after uni-Nx to nadir about 50% below baseline but then increased back to baseline by 10-days post-uni-Nx when compensatory kidney growth had been completed. Therefore, the 2-day post-uni-Nx peak of urine IGF-1 excretion corresponded to the 2-day peak of maximal kidney growth rate, consistent with IGF-1 serving as a potential driver of compensatory kidney growth.

#### IGF-1 receptor (IGF-1R) inhibition by picropodophyllin (PPP) reduces post-Nx compensatory kidney hypertrophy

Haylor et al. previously reported that a cyclic peptide IGF-1R antagonist reduced post-Uni-Nx compensatory kidney growth, thereby providing direct evidence that compensatory kidney hypertrophy is driven through the IGF-1R^32^. To confirm this result we tested the effect of the small molecule competitive inhibitor PPP designed to target the tyrosine kinase receptor^35^. Actively growing 100 g rats were used so that the effect of PPP to inhibit normal body growth through IGF-1 and IGF1R could serve as an internal positive control for drug efficacy. Data are shown in Fig. 3C. PPP administered IP every 12 h starting 24 h before uni-Nx and continued for 8 days after uni-Nx significantly reduced body weight gain from 27.0 ± 2.5% to 16.7 ± 4.6% (*P* < 0.01), (a 38% reduction compared to vehicle), thereby confirming the PPP efficacy under the experimental conditions used (Fig. 2C, left panel). Following uni-Nx, kidney weight in vehicle-treated rats increased 56.5 ± 8.2% over the 8 day period of observation representing the combination of normal kidney growth (that is proportional to body growth) added to compensatory kidney hypertrophy (in response to the uni-Nx). In PPP-treated rats, kidney weight increased 33.9 ± 5.3%, a 40% reduction compared to vehicle-treated rats (*P* < 0.01) (Fig. 3C, middle panel). The compensatory kidney growth component was assessed by the kidney weight:body weight (Kw:Bw) ratio. The Kw/Bw ratio in vehicle-treated rats increased 23.2 ± 5.3% compared to that in PPP-treated rats which increased 14.8 ± 2.1% (*P* < 0.05), a 36% decrease (Fig. 3C, right panel). Thus, the effect of PPP to decrease body growth (serving as a positive internal control for IGF-1R inhibition) was comparable to the reduction in compensatory kidney hypertrophy, consistent with body weight gain and compensatory kidney hypertrophy both being driven in part via the IGF-1R.

#### Post-uni-Nx 2-day urine IGF-1 peak was reduced by ACEi treatment initiated before uni-Nx

**Figure 4:**
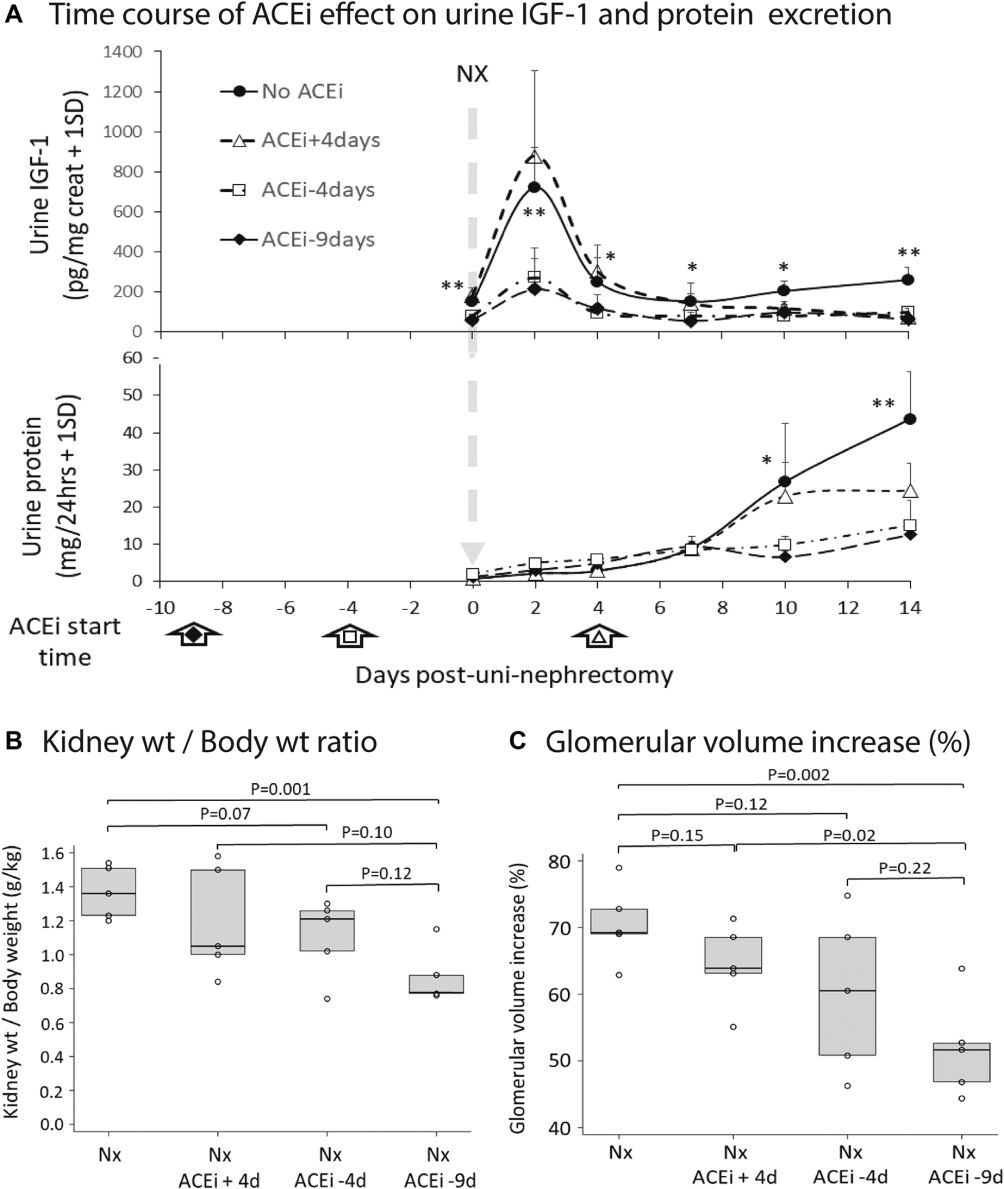
Short-term time-course post-uni-Nx showing the effect of ACEi started either 4 days *after* uni-nephrectomy or 4 or 9 days *before* uni-nephrectomy. Panel (**A**) Upper Panel. Effect of ACEi timing on urine GF-1 excretion. ACEi (enalapril 10 mg/kg/day in drinking water) was started 9 days before uni-Nx (solid triangles) or 4 days before uni-Nx (open squares) significantly reduced the baseline IGF-1 excretion, the 2-day IGF-1 peak of urine IGF-1 following uni-Nx, and urine IGF-1 excretion remained lower than control non-ACEi-treated rats by day-14. If ACEi was started 4-days post-uni-Nx, the 2-day urine peak was preserved, but by day-14, the urine IGF-1 had decreased to levels similar to the ACEi pre-Nx treated groups. N = 5 per group. *P < 0.05, **P < 0.01. Panel (**A**) Lower Panel. Effect of ACEi timing on 24 h urine protein. Urine protein excretion in non-ACEi-treated rats did not increase significantly following uni-Nx until day-10 and 14. ACEi pre-treatment significantly reduced urine protein at day-10 and 14 post-uni-Nx. By day-2 following uni-Nx, when IGF-1 had increased 8-fold, urine protein excretion was not increased. Panel (**B**) Effect of ACEi pre-treatment on the kidney weight/body weight ratio by day-14 post-uni-Nx. Panel (**C**) Effect of ACEi pre-treatment on the glomerular volume by day-14 post-uni-Nx. Kidney weight/body weight ratio and glomerular volume increase were significantly reduced compared to the non-ACEi-treated group by ACEi started 9-days before uni-Nx. ACEi started 4-days before uni-Nx did not reach statistical significance. The error bars are 1SD. *P < 0.05, **P < 0.01.

As shown in Fig. 4A upper panel, if ACEi was begun either nine days or four days *before* uni-Nx, the two-day peak of IGF-1 appearing in urine was significantly reduced. In contrast, as expected, if the ACEi was initiated four days *after* uni-Nx, the day-2 post-uni-Nx IGF-1 peak was not affected, although by day 14 after uni-Nx urine IGF-1 had decreased to the same extent as was observed if ACEi initiated before uni-Nx. Figure 3A lower panel shows 24 h urine protein values in the same urine samples. Notably, by day-2 post-uni-Nx there was no measured increase in urine protein excretion associated with the day-2 IGF-1 peak in either ACEi-treated or non-ACEi-treated rats, although by day-14 post-uni-Nx when kidney size had increased (shown in Fig. 2A), the 24 h urine protein in non-ACEi-treated rats had significantly increased.

#### ACEi initiated nine days before uni-Nx, but not four days after uni-Nx, reduced compensatory kidney and glomerular hypertrophy in the remaining kidney

Figure 4B and C show that ACEi started nine days before uni-Nx significantly reduced both compensatory kidney weight increase (*P* = 0.001) and GV increase (*P* = 0.002) by 14 days post-uni-Nx. If ACEi was started four days before uni-Nx, both kidney weight and GV were reduced at 14-days post-uni-Nx, but did not reach statistical significance. If ACEi was initiated four days after uni-Nx, no reduction in kidney weight or GV was observed at 14-days post-Nx. Thus, the rapid onset of growth in the remaining kidney and glomerulus after uni-Nx was significantly inhibited by ACEi treatment if initiated 9-days before uni-Nx.

#### Blood IGF-1 was decreased 2-days post-uni-Nx

Serum IGF-1 decreased from 27.5 ± 12.9 ng/mg protein on day of uni-Nx to 5.0 ± 1.3 ng/mg protein on day-2 post-uni-Nx (n = 5, *P* < 0.05), consistent with flux of IGF-1 from the blood into the kidney after uni-Nx. In contrast, in rats started on ACEi 9-days before uni-Nx, the serum IGF-1 level was 37.5 ± 15.7 ng/mg protein on the day of uni-Nx and 26.4 ± 4.4 ng/mg protein on day-2 following uni-Nx (n = 5, *P* = 0.30), consistent with an ACEi-induced reduction in IGF-1 flux from the blood into the remaining kidney.

## Discussion

Using transgenic rat models we previously reported that glomeruli can become destabilized by podocyte depletion leading to autonomous progression to ESKD that is triggered and driven by critical reduction in podocyte density achieved either by specific reduction in podocyte number or by increased GV induced by uni-nephrectomy^25,26,36,37^. In the current study, using wild type rats with normal podocytes, we confirmed that uni-nephrectomy-induced glomerular enlargement also resulted in autonomous progressive glomerulosclerosis in the absence of overt hypertension. Therefore, to learn how to maintain podocyte density to preserve renal function, we focused on how post-nephrectomy glomerular enlargement could be slowed or prevented by ACEi.

A key finding presented in this report is the observation that the timing of ACEi initiation in relation to the uni-nephrectomy had a significant impact on both short-term and long-term GV increase. This is consistent with the hypothesis that peri-nephrectomy or, by extension peri-transplant, hemodynamic events (pressures and/or flows) are critically important to determining long-term single kidney health. Prior reports documenting higher filtration fraction in larger body size allograft recipients as well as the well-known relationship of kidney size mismatch to long-term kidney allograft outcome is also consistent with this hypothesis^14,15^.

Since there is substantial data supporting the role for IGF-1 in post-Uni-Nx compensatory growth of the remaining kidney, we examined urine IGF-1 as a readout that could potentially be used to monitor this process^29–31^. Our data is also consistent with the hypothesis that post-uni-Nx hyperfiltration is associated with increased filtration of IGF-1 (detectable in the urine) that in turn drives glomerular enlargement triggering critical podocyte depletion and downstream consequences, including proteinuria and glomerulosclerosis. We emphasize that other factors hyperfiltered from the blood, such as growth hormone and IGF-2, and effects associated with hyperfiltration on glomerular hemodynamics, shear stress, tubular cilial sensing, and other mechanisms, will likely also contribute to regulating compensatory kidney and glomerular growth. However, urinary IGF-1 can potentially serve as a useful marker of hyperfiltration-induced kidney exposure to growth factors.

These data also provide a possible road map for how nephrectomy-induced hypertrophic stresses could be mitigated with the goal of prolonging single kidney lifespan. While an IGF-1 receptor blocker would be expected to prevent glomerular hypertrophic stress, most cells express IGF-1 receptors so that IGF1 receptor blockade would likely have significant off-target side-effects that would limit long-term use. In contrast, the effect of ACEi is to reduce kidney hyperfiltration and thereby selectively reduce excess IGF-1 (and other potential hyperfiltered factors which may drive compensatory hypertrophy) delivered into the kidney. Thus ACEi, an inexpensive medication for which extensive clinical experience is already available, can be viewed as selectively reducing excess kidney IGF-1 exposure.

The ACEi timing experiment shows that ACEi started as early as four days after uni-Nx led to only a partial reduction of GV enlargement. This may explain in part why studies of ACEi initiated weeks or months after kidney transplantation have failed to preserve allograft function. In contrast, a recent report from Cockfield et al. starting angiotensin II blockade early after transplantation was associated with significantly improved histology^38–40^. Optimally prevention of compensatory glomerular enlargement may require ACEi to be initiated several days before uni-Nx. A potential concern for this strategy is that reducing normal compensatory kidney and glomerular hypertrophy might significantly impair allograft function for the long-term. Notably, in long-term studies we did not observe a measurable effect of starting ACEi more than 7-days before uni-nephrectomy on creatinine clearance or kidney weight even although compensatory GV increase was largely prevented. Thus, this theoretical concern may not prove to be prohibitive.

In summary, accumulating data suggests that shorter kidney donor and allograft lifespan may be a predictable phenotype of the single kidney state*.* If correct, then extending single kidney lifespans will require active intervention to mitigate underlying hypertrophic stress mechanisms incurred at time of nephrectomy and/or transplantation. Based on modeling, these stresses might be prevented by ACEi treatment started before or very early after nephrectomy or transplantation. Further studies are required to determine whether these strategies do improve outcomes for single kidney states, and whether they can be safely used in man.
